# Application of a Custom Device to Measure Isometric Knee Strength: Possible Injury Correlation in Professional Soccer (Football) Players

**DOI:** 10.3390/jfmk8040141

**Published:** 2023-10-05

**Authors:** Paolo Cigni, Tommaso Minuti, Andrea Mannini, Alessandro Cucini, Michele Costagli, Stefano Rapetti, Luca Alimonta, Erika Cione, Roberto Cannataro, Leonardo Ricotti

**Affiliations:** 1 Auxilium Vitae Volterra Spa, Borgo San Lazzaro 5, 56048 Volterra, PI, Italy; paolocigni@tiscali.it; 2 Italian Society of Nutrition Sports and Well-Being (SINSeB), Via Luigi Cherubini, 2, 50053 Empoli, FI, Italy; 3 Fisioclinic Dott. Paolo Cigni, Via Porta Massetana 1, 56045 Pomarance, PI, Italy; ale.cucini@gmail.com (A.C.); costagli.michele@gmail.com (M.C.); 4 The BioRobotics Institute, Scuola Superiore Sant’Anna, 56127 Pisa, Italy; tommaso.minuti@gmail.com (T.M.); leonardo.ricotti@santannapisa.it (L.R.); 5 Department of Excellence in Robotics & AI, Scuola Superiore Sant’Anna, 56127 Pisa, Italy; 6 IRCCS Fondazione Don Carlo Gnocchi ONLUS, 50143 Firenze, Italy; amannini@dongnocchi.it; 7 A.C. Milan, Via Aldo Rossi 8, 20149 Milano, Italy; nebulin@libero.it; 8 Sporting Club Madonna di Campiglio, Via Monte Spinale 16, 38036 Madonna di Campiglio, TN, Italy; luca.alimonta@gmail.com; 9 Department of Pharmacy, Health and Nutritional Sciences, University of Calabria, 87036 Rende, CS, Italy; 10 Galascreen Laboratories, University of Calabria, 87036 Rende, CS, Italy; 11 Research Division, Dynamical Business & Science Society—DBSS International SAS, Bogotá 110311, Colombia

**Keywords:** muscle strength assessment, dynamometer, knee extensor, knee flexor, sports injuries, soccer injuries, hamstring, strength training

## Abstract

Injury in sports is an occurrence that prevents athletes from participating in training and competitions and has an incidence of 8.1 injuries/1000 h of practice. This translates into a cost and also into danger, especially if the event is repeated, for the health of the athlete; the injury certainly has a multifactorial causality. On the other hand, having instruments that can represent an alarm could be helpful for those involved in sports science. We used a specifically designed instrument, presented in a previous work, which shows excellent reliability and repeatability in measuring the strength of the knee flexors and extensors to test 107 players belonging to three different teams playing in the Italian Serie A. We took three measurements, beginning of the season, mid-season, and close to the end of the season. This retrospective study on 107 professional soccer players demonstrates that isometric force-related parameters of the knee extensors and flexors are associated with the risk of injury to lower limbs. Logistic regression evidenced a significant correlation between the parameter indicating the imbalance of the force between the flexors of the two limbs (p≤0.05, OR = 1.089) and the occurrence of injuries. Survival analyses (p≤0.001) evidenced a correlation between the population survival time and the injury incidence. We demonstrated that the analysis of the strength imbalance is correlated with injury occurrence, but it is well known that sports injuries are a multifactorial event; so, they cannot be predicted by only one parameter. However, the method proposed in this paper could represent a useful tool for sport scientists.

## 1. Introduction

Among team sports, soccer is one of the most prone to produce injuries [1]. According to a study promoted by UEFA, 87% of soccer injuries involve the lower limbs, of which 17% concern muscle injuries (12% flexors and 5% extensors) and 5% trauma to the anterior cruciate ligament (ACL) [2]. Several studies [3,4,5] have analyzed the possible factors increasing the risk of injury, classifying them into nonmodifiable risk factors (e.g., age, height, weight, previous injuries, etc.) and modifiable risk factors (e.g., strength, fatigue, flexibility, etc.). Strategies to predict and prevent injuries are of great interest to professional soccer teams [6]. Muscle strength is responsible for joint torque production. It is crucial in determining the efficacy of human body movements and guaranteeing joint stability and posture maintenance [7]. Muscle strength has been the object of many studies in which the relationship between muscle strength, training, level of competition, and performance has been explored [8,9,10]. The role of muscle strength deficits as possible risk factors for lower limb injuries represents a debated topic. Recent studies showed that hamstring and quadriceps weakness and imbalances did not correlate with injuries [3,11].

Nevertheless, evidence suggesting that muscle imbalances play an important role is recently accumulating in many sports. Thorlund et al. demonstrated that a lower hamstring-to-quadriceps muscle activation level was correlated with ACL injury in handball and soccer players [12]. Furthermore, Croisier et al. found that restoring the balance between agonist and antagonist muscle groups considerably decreased the risk of injuries [13]. Drigny and colleagues reported that muscle strength imbalances between the internal and external rotators of the shoulder were associated with an increased risk of shoulder injuries during the season in adolescent swimmers [14]. Knee injuries are common in runners but also in other sports including soccer. Some authors suggested that some pathologies of the knee, e.g., the patellofemoral pain syndrome, the iliotibial band friction syndrome, and the patellar tendinopathy, are triggered by muscle imbalances. In this context, different theories exist; for example, considering the patellofemoral pain syndrome, a theory postulates that patellar maltracking is mainly caused by the weakness of the vastus medialis obliquus, which introduces patellar tilting due to overpowered lateral structures, including the iliotibial band, lateral retinaculum, and vastus lateralis that compensate for such weakness. Another theory suggests that decreased eccentric hip abductor and external rotator strength cause a relative femoral internal rotation and/or hip adduction moment, with consequent increased compressive forces of the patella in the trochlear groove during dynamic movements [15]. On soccer players, Rahnama et al. observed that muscular imbalance between the preferred and non-preferred leg in sub-elite football players can be an injury risk factor [16]. Strength imbalance has also been identified as a general risk factor for hamstring injuries [17]. Indeed, based on these findings and similar ones, Pérez-Gómez et al. recently reviewed the physical exercises that can be used for preventing injuries among adult male football players, showing that some of them are based on muscle strengthening and re-balance [18]. Overall, these hints suggest that this topic deserves to be investigated further.

It must be considered that the nature of injuries, especially in soccer, is multifactorial. Consequently, it is almost impossible to find a test describing the whole variance of this problem and fully predicting it [5,19,20]. However, finding a screening methodology having a certain degree of correlation with the onset of injuries (although not describing the whole variance) would be very useful for professional soccer teams to: (i) monitor the athletic condition of a player over time, (ii) guide the recovery process from an injury, and (iii) early identify specific deficits in strength potentially increasing injury risks and correcting them with ad hoc training. In the state of the art, considerable attention has been devoted to isokinetic strength assessment. Isometric force measurements have yet to be explored for this purpose. Both measurements provide helpful information. Isokinetic strength gives information on the dynamic qualities of the muscle tested.

In contrast, isometric tests provide the maximal voluntary isometric contraction the muscle can produce [21,22]. In this study, we focused on isometric force assessment, underexplored in soccer, as a possible screening methodology to correlate with the onset of injuries. Based on manual resistance, handheld dynamometers (HHD) are among the most used systems for measuring isometric force. They show moderate to excellent reliability [23,24]. These devices are affected by relatively high variability and subjectivity of measurement [25]: the reliability varies considerably, depending on the muscle action tested [26]. Their use is questionable for these reasons, mainly when a slight deficit or imbalance in knee muscle strength is targeted. Due to such a lack of reliability, only a few studies aimed to find an association between isometric strength indices, assessed with HHD, and injury occurrence [27,28,29]. More recently, dynamometer anchoring systems [30,31,32] were developed to measure the isometric strength of lower limbs. The anchored systems have shown increased reliability compared to HDD. However, no studies have been conducted to evaluate the association between the maximum voluntary isometric contraction (MVIC) values measured with these devices and lower limb injuries.

## 2. Materials and Methods

**Figure 1:**
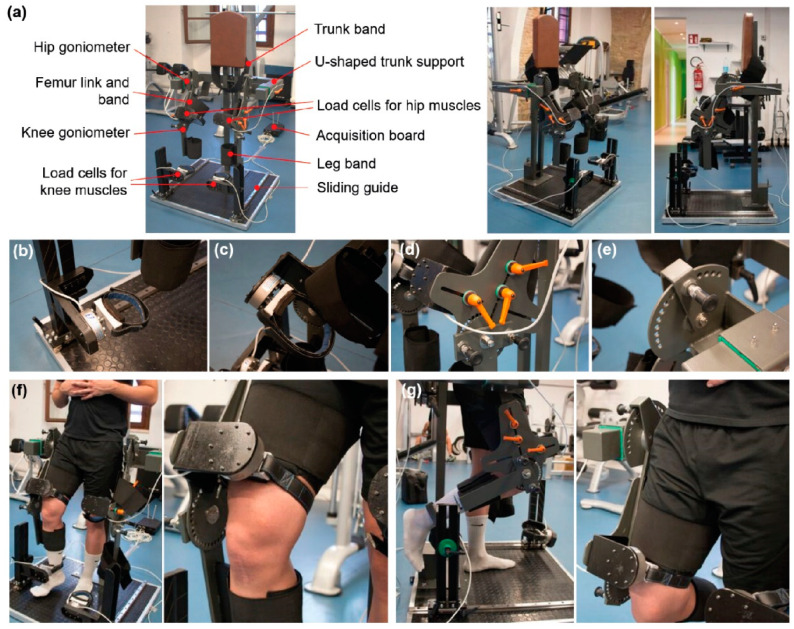
Description of the custom device to measure the isometric force used in this study. Scheme of the instrument with the critical components highlighted and pictures of the device from different viewpoints (**a**). Zoomed views highlight some essential components of the platform: load cell at the ankle (**b**); load cells at the knee (**c**); handles allowing one to adapt the machine dimensions to the subject anatomical characteristics (**d**); and goniometer to verify the flexion/extension degree of the joint (**e**). (**f**) Images of a subject undergoing a knee extensor force measurement test; (**g**) images of a subject undergoing a knee flexor force measurement test. Reprinted from [33].

For strength assessment, we used a device composed of an exoskeleton that keeps the leg in a fixed position (regarding angles), and it is remarkably reliable; the whole device and the testing procedure was shown in our previous work [33], and the device is shown in Figure 1 and Table S1 (Supplementary Material).

Our protocol involved 107 professional soccer players enrolled in three professional teams (age: 25.50 ± 7.78 years, height: 185 ± 1.00 m, and body mass: 78.00 ± 2.82 kg). Athletes playing in all roles (including goalkeepers) were included in the study. Demographic and anthropometric data of the age, weight, height, BMI, and the role of players were collected at the beginning of the season to include nonmodifiable factors for possible injury occurrence (Table 1) [34].

Subjects were excluded from the study if they felt pain during the preliminary mock test. Moreover, athletes presenting a scar or a wound at or near the support of the load cell were also excluded.

The investigation was conducted in accordance with the Declaration of Helsinki. Institutional ethical approval was granted. Before taking part in the study, all subjects were informed of the benefits and risks, and informed written consent was subsequently obtained.

The study was conducted over the seasons, and the test was repeated at three different time-points in each season: an initial one (in August), an intermediate one (in November), and a final one (in March). 

All subjects underwent at least one test; but it was possible that the test was administered up to 3 times. The observational time to assess injuries was 90 days.

A standard injury registration procedure was used to report the mechanism that led to the damage. A soccer player was considered injured if the trauma forced him to miss at least one official game. At the same time, he was defined as recovered when he could perform complete training with the rest of the group and was available to play an official match. In the months following the test, each team provided the authors with a detailed report specifying all the players who were injured in that period, the location of the injury, the injury date, and how many days the athlete took to recover from the injury. In this study, we focused on three types of injuries physiologically related to the knee flexor and extensor strength: muscle injuries to the flexors and extensors and ACL traumas.

The device provided the maximum force values for the knee extensors ($E_{r};E_{l\;}$) and the maximum force values for the knee flexors ($F_{r};F_{l\;}$) of both limbs (r = right; l = left). In addition, the corresponding activation times ($T_{1};\;T_{2};\;T_{3};\;T_{4})$ were recorded. The activation time consisted of the time needed by the subject to reach 60% of the maximum force value. 

For each player, the strength relative to body mass index (BMI) was evaluated by calculating the ratio between the isometric knee muscle strength and the BMI of the subject. Such normalization is widely used in the state-of-the-art [34] to make force values more comparable to each other, considering the subject anthropometric variability. 

Specific combinations of these parameters were devised in order to to have indications of athletes’ muscle imbalances between their limbs. The “bilateral ratio” of flexors (Flexor Bilateral Ratio, FBR) or extensors (Extensor Bilateral Ratio, EBR) compared the maximum force of the same muscle group between two limbs. The “unilateral ratio” of the right limb (Right Unilateral Ratio, RUR) and left limb (Left Unilateral Ratio, LUR) compared the maximum force of two antagonist muscle groups for the same leg. These parameters were calculated as follows:

(1) $$EBR=\frac{\left(E_{r}-E_{l}\right)*100}{E_{r}},$$

(2) $$FBR=\frac{\left(F_{r}-F_{l}\right)*100}{F_{r}},$$

(3) $$RUR=\frac{\left(E_{r}-F_{r}\right)*100}{E_{r}},$$

(4) $$LUR=\frac{\left(E_{l}-F_{l}\right)*100}{E_{l}}.$$

After each test, the results obtained were shared with the athletic trainers of the soccer teams, also suggesting who were the players at “risk of injury”. A player was considered at risk of injury if he presented one or more of these factors (pathological cutoffs): bilateral imbalance higher than 18% (EBR and FBR > 18); unilateral imbalance higher than 40% (RUR and LUR > 40); maximum extensor strength value normalized on the BMI lower than 20 ($E_{r}/BMI$ and $E_{l}/BMI$ < 20); maximum flexor strength value normalized on the BMI lower than 15 ($F_{r}/BMI$ and $F_{l}/BMI$ < 15). 

Two parallel analyses were carried out: (1) a logistic regression adjusted by age identified the predictors of injury from the available measurements (retrospective analysis), and (2) a survival analysis was conducted to understand whether the injury occurrence was correlated with risk factors calculated from the tested parameters. 

The tested variables for both analyses were the ones indicating possible muscle weaknesses or muscle imbalances, namely: $E_{r},\;T_{1},\;E_{r}/BMI,E_{l\;},\;T_{2},\;E_{l}/BMI,F_{r},\;T_{3},\;F_{r}/BMI,{\;F}_{l\;}$,$T_{4}$,${\;F}_{l}/BMI,EBR$, FBR, RUR, and LUR, together with the nonmodifiable variables.

A multivariate binomial logistic regression adjusted by age was performed to ascertain the combined effect of all groups of variables that showed an even weak significance in the univariate tests concerning injury prediction. This method used the dichotomous variable indicating injury (1 = injured athlete; 0 = non-injured athlete) as the dependent one. The continuous variables’ linearity for the dependent variable’s logit was assessed using the Box–Tidwell procedure, and multicollinearity was excluded by verifying that the variance inflation factors were lower than five for all variables. The effect size of the independent prognostic factors was expressed through the odds ratios, with the respective 95% Confidence Interval (CIs). We tested our logistic regression models for predictive accuracy using Nagelkerke’s R^2^. 

A Cox regression was then used for analyzing injury timing with respect to the exposure to risk factors. Risk factors were derived from the available variables using the pathological cutoffs described in the previous paragraph. Athletes were censored when a test was repeated before the observation time (190 days), when a player moved to another club and was no longer monitored, or when the player suffered a different kind of injury with respect to the ones on which this study is focused, for which it was not correct to assume that the muscle strength remained unchanged.

After this analysis, receiving operating characteristics (ROC) curves were evaluated for each variable involved in the multivariate logistic regression. This analysis was conducted to obtain insights into the relevance of the single risk factors and to select the optimal thresholds for risk assessment, as identified by available data. The achieved data-driven thresholds were then compared with the previously mentioned pathological cutoff, which the authors selected a priori. 

All analyses were performed using SPSS 26.0 software (SPSS, Chicago, IL, USA) running on Windows. The significance level was set in all mentioned analyses at a *p*-value < 0.05.

## 3. Results

### 3.1. Association between Isometric Force and Injuries: Results of the Logistic Regression 

The first step in the analysis of the 107 professional soccer players consisted of the implementation of a univariate logistic regression. This allowed selecting the demographic variables (associated with lower limb injuries in terms of Odds ratio (OR) (Table 2). Similarly, univariate logistic regression was performed for strength parameters (Table 3). 

Age, height weight, BMI, and player position showed no correlation with the probability of injuries [35] highlighting that some strength parameters ($E_{r},$ $E_{r}/BMI$, $F_{r}$, $F_{r}/BMI$, FBR, and RUR) were significantly (*p* < 0.05) associated with the injuries recorded in professional soccer players. Moreover, other parameters ($E_{l}$, $\frac{E_{l}}{BMI},\;EBR,andLUR$) were weakly associated (*p* < 0.15) with the injury risk. By including the significant or weakly significant variables in a multivariate model adjusted by age, the resulting solution explained about 32% of the variance of the problem (Nagelkerke’s $\;R^{2}\;$= 0.322) with satisfactory specificity (100%) and sufficient sensitivity (31.8%) (Table 3).

### 3.2. Association between Isometric Force and Injuries: Survival Analysis

A Cox regression was computed using all the strength variables, remapped to dichotomous variables using pathological cutoffs as indicated by the therapists to discriminate players at risk of injury (Supplementary Table S2). The results of this multivariate survival analysis proved that the occurrence of the injury was correlated (p≤0.001) with the time of the observation of the event.

**Figure 2:**
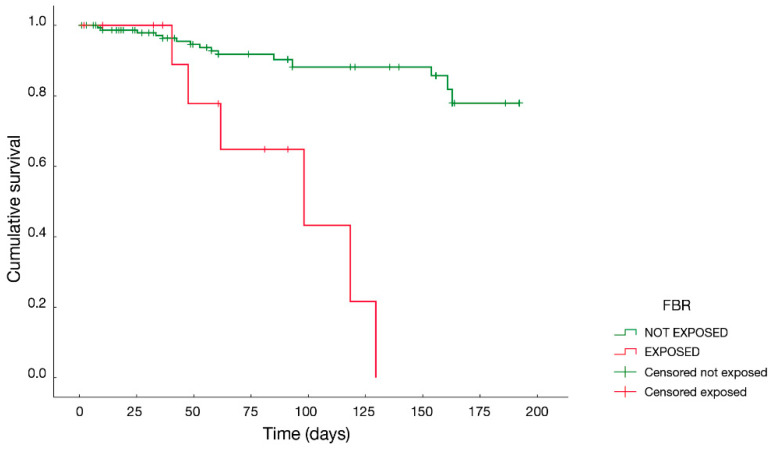
Survival curve of the FBR parameter. The “exposed” group consists of subjects in which the force imbalance between the flexors of the two limbs is higher than 18%. The “not exposed” group includes the subjects not considered at risk of injury, concerning flexor imbalance.

The FBR was the parameter for which the association with injuries was more evident, with an observed risk of 5.16, as shown in Figure 2.

**Figure 3:**
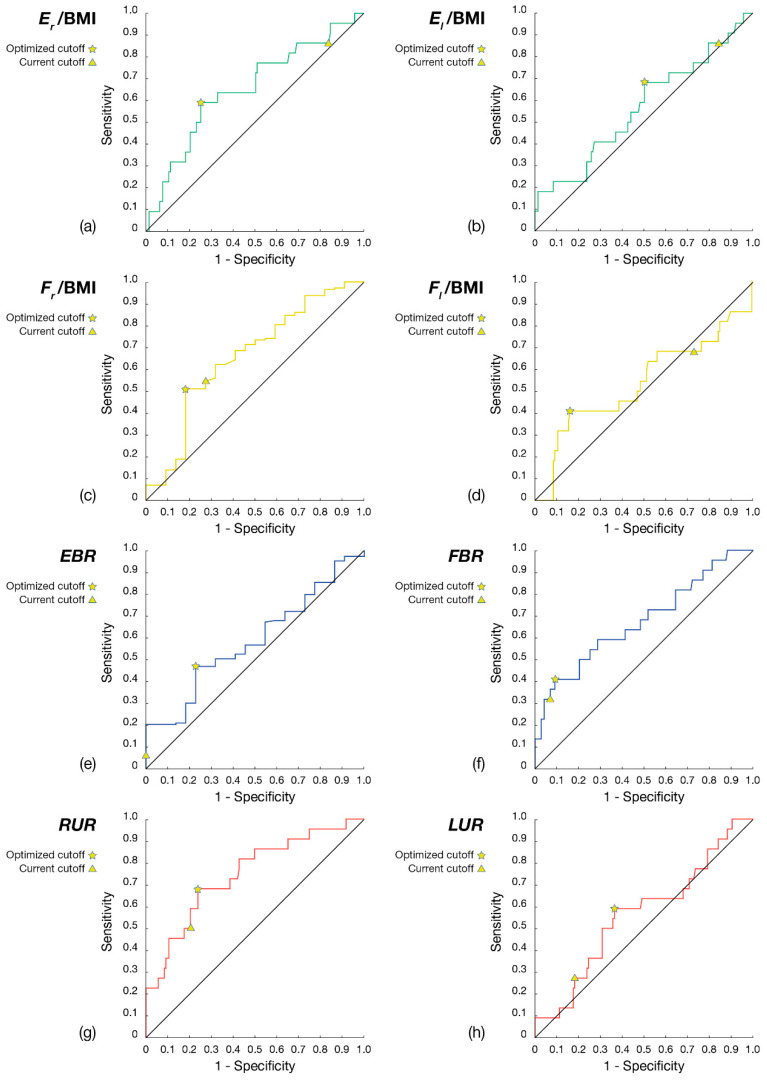
ROC curves of the parameters included in the multivariate analysis, with current and optimized cutoffs highlighted on the graphs: (**a**) E_r_/BMI; (**b**) E_l_/BMI; (**c**) F_r_/BMI; (**d**) F_l_/BMI; (**e**) EBR; (**f**) FBR; (**g**) RUR; (**h**) LUR.

The cutoff values that were adopted by the therapists to select players at risk of injury and described in the previous sections were optimized, building on available experimental data and developing a series of ROC curves, as shown in Figure 3a–h and in Table S3.

## 4. Discussion

Remarkably, the results of the retrospective study on 107 professional soccer players demonstrate that isometric force-related parameters of the knee extensors and flexors are associated with the risk of injury to lower limbs. 

Age was not associated with injury occurrence. This result is in contrast with some works [11,35,36]. For example, Van Dyk and colleagues observed a 7% increased risk of hamstring injury per year added; the authors stated that the injury risk of the youngest group (<22 years) was 85% less than the one of other (older) groups. However, other studies did not find a correlation between age and injuries [37,38]. In any case, to reduce the risk of introducing a bias in our results, we adjusted the multivariate model by age.

In our case, BMI, height, weight, and player position also did not show an association with the injury risk. These results are in line with previous works [11,30,38,39,40].

The univariate analysis on strength-related parameters highlighted that the FBR (Flexors Bilateral Ratio), related to an imbalance in the flexor strength, is a significant injury risk factor for professional male soccer players [41,42].

Injury is a relatively rare event. First statistical significances were achieved in this study from logistic and Cox regression analyses. This is encouraging to suggest our solution and some of the measured quantities for clinical trials to come. A post hoc power analysis at this level would be inappropriate [43,44,45]. However, our results will enable researchers to conduct more accurate power analyses in future clinical evolutions of this study.

Although our results confirm the reliability and usefulness of this test for a professional soccer team, it must be underlined that it cannot be considered an injury prediction screening test. In fact, as reported by Bahr [14], if a significant association is identified between one or more risk factors and sports injuries, this represents only the first of three steps to validate a screening program. These steps include: (1) the definition of rules to separate the athletes at high risk of injury from the other ones; (2) then, these rules must be applied to a new population of subjects, and the method predictive power must be observed, thus validating the method; (3) finally, a randomized controlled trial must be carried out, in which the injury rate of a treatment group is compared with the one of a control group.

Correira et al. recorded maximal isometric knee extension and flexion strength of twenty-four professional football players (12 uninjured and 12 injured in knee flexors) at static hip and knee angles of respectively 85° and 30° [29]. They selected this knee position to correspond with the peak hamstring elongation during the terminal swing phase in high-speed running. Their results showed no correlation between the hamstrings to quadriceps imbalance index or their MVIC values and hamstrings injuries. This may suggest that the swing position may not be ideal for testing the maximum force of these muscles. Likewise, no significant results were shown by Gossens using an HHD to acquire the MVIC of quadriceps in a sitting position with the knee flexed at 60° and flexors in a supine position with the knee flexed at 30° [27]. 

A novel apparatus to measure knee flexor isometric strength was used by Hickey et al. in a retrospective study on the occurrence of hamstring injuries to evaluate its correlation with isometric strength parameters [28]. They observed that subjects who suffered a flexor injury showed a bilateral flexor imbalance compared to those who were injured, in accordance with our results.

Croisier and colleagues pointed out the importance of monitoring strength multiple times over a season, instead of taking a single measurement (e.g., at the beginning of the season) [13]. In our study, we took this aspect into consideration, differently from many other studies, by assessing strength in three time-points over the season and monitoring two different seasons. The survival curve of the portion of the population having an imbalance of the flexors (FBR greater than 18%) was compared to the one showing no imbalance. The subjects presenting such a muscular imbalance had a significantly shorter average injury time than the other ones. This result shows the high potential of using such an index to evaluate the injury risk in soccer players and constitutes an original finding in the state of the art of isometric force screening.

It is worth pointing out that the specific strength training program administered to the players considered at risk of injury represents a factor that reduced the possibility to find a significant association between the injury event and the strength-related parameters. In fact, thanks to such a corrective program, the athlete improved his strength performance, thus reducing the injury risk with respect to a condition in which the test would have been performed without communicating the result to the athlete and the trainers. This makes even more relevant the fact that a statistically significant association was found in this study. Probably, performing the test and collecting data without sharing them with the athlete and the trainers (something that is difficult to achieve with high-level professional teams) could lead to even more significant associations. This can be considered a future perspective of this study.

The computation of ROC curves maximized the sensitivity and specificity of the model. Based on these results, a soccer player should be considered at risk of injury when: (1) the ratio between the force of the right extensor (or flexor) and the left one is greater than 18%, or (2) the imbalance between the strength of the two antagonist muscle groups of the same limb is greater than 40%, or (3) when the muscle group expresses a force value, normalized for the BMI of the subject, lower than 20 for the extensors or 15 for the flexors. 

As a possible future perspective, these optimized cutoffs may be used to test the predictive power on a new group of professional male soccer players or insert them in a process of cross validation.

## 5. Conclusions

The method proposed in this work, based on isometric force assessment and evaluation of muscle imbalances, proved to be an exciting tool helping to predict injury events. The test had high reliability (because the position is fixed, and it is carried out at an optimal angle). A correlation between the flexor bilateral ratio and injury occurrence was found. Other correlations with injury also emerged, in particular for the strength of the right extensor normalized by the body mass index and the imbalance between flexors and extensors of the right limb. It must be underlined, as a limitation, that the study is observational. Therefore, it is not randomized or controlled. Future studies will be necessary to consolidate this type of tool and method. The future standardization of this test would make it more robust and usable in the field of sports science but also in the medical field, for example, for the management of sarcopenia [46].

## Figures and Tables

**Table 1:** Demographic data of the soccer players involved in the study.

	Injured (*n* = 22)	Uninjured (*n* = 85)
Anthropometric data (mean ± SD)		
Age, years	25.54 (±4.72)	25.57 (±5.04)
Height, m	1.82 (±0.06)	1.83 (±0.07)
Weight, kg	78.92 (±6.84)	78.15 (±6.74)
BMI, kg/m	23.65 (±1.76)	22.98 (±2.78)
Player position		
Goalkeeper	0 (0%)	11 (12.95%)
Defender	9 (40.91%)	28 (32.94%)
Midfielder	6 (27.27%)	28 (32.94%)
Forward	7 (31.82%)	18 (21.17%)

**Table 2:** Results of the univariate logistic regression for demographic variables.

	Regression Coefficients (B)	Standard Error	Wald Test	*p*-Value	Odds Ratio (OR)	OR Confidence Interval 95%		$R^{2}$ Nagelkerke
						Lower	Upper	
Age	−0.01	0.04	0.01	0.92	0.99	0.91	1.08	0.01
Height	−1.41	3.69	0.14	0.71	0.25	0.01	2.21	0.01
Weight	0.02	0.04	0.23	0.63	1.02	0.95	1.09	0.01
BMI	0.21	0.17	1.52	0.22	1.24	0.88	1.73	0.03
Player position	0.31	0.26	1.46	0.23	1.37	0.82	2.28	0.21

**Table 3:** Results of the univariate logistic regression for strength parameters.

		Regression Coefficients (B)	Standard Error	Wald Test	*p*-Value	Odds Ratio (OR)	OR Confidence Interval 95%		$R^{2}$ Nagelkerke
							Lower	Upper	
Right extensor	$E_{r}$	0.01	0.01	4.74	0.03 *	1.00	1.00	1.01	0.05
	$T_{1}$	−1.92	2.65	0.52	0.47	0.14	0.01	26.83	0.01
	$\frac{E_{r}}{BMI}$	0.12	0.05	4.44	0.03 *	1.13	1.01	1.27	0.05
Left extensor	$E_{l}$	0.01	0.01	3.03	0.08	1.00	1.00	1.01	0.03
	$T_{2}$	2.56	2.27	1.27	0.26	13.01	0.15	1130.26	0.01
	$\frac{E_{l}}{BMI}$	0.09	0.05	2.50	0.11	1.09	0.98	1.23	0.02
Right flexor	$F_{r}$	−0.01	0.01	4.97	0.03 *	0.99	0.98	1.00	0.05
	$T_{3}$	−0.11	0.41	0.07	0.77	0.89	0.39	2.01	0.01
	$\frac{F_{r}}{BMI}$	−0.19	0.07	6.24	0.01 *	0.82	0.71	0.959	0.07
Left flexor	$F_{l}$	0.01	0.00	0.01	0.92	1.00	0.99	1.01	0.01
	$T_{4}$	0.39	1.67	0.05	0.81	1.48	0.06	39.99	0.01
	$\frac{F_{l}}{BMI}$	0.01	0.06	0.01	0.98	1.00	0.87	1.14	0.01
Muscle imbalances	EBR	−0.06	0.04	2.20	0.13	0.93	0.86	1.02	0.02
	FBR	0.09	0.03	11.79	0.01 *	1.10	1.04	1.16	0.10
	RUR	0.08	0.02	14.39	0.01 *	1.08	1.04	1.13	0.19
	LUR	0.03	0.02	2.68	0.10	1.03	0.99	1.07	0.03

*: Statistically different values.

## Data Availability

Data are available under request from P.C.

## Supplementary Materials

The following supporting information can be downloaded at: https://www.mdpi.com/article/10.3390/jfmk8040141/s1
