# Supplementary material for: Application of a Custom Device to Measure Isometric Knee Strength: Possible Injury Correlation in Professional Soccer (Football) Players
Source: J Funct Morphol Kinesiol. 2023 Oct 5;8(4):141. doi: 10.3390/jfmk8040141 (PMC10594464; doi:10.3390/jfmk8040141)
Supplement: Supplementary file 1 [file jfmk-08-00141-s001.zip › jfmk-2572156-supplementary.pdf]

Table S1. Results of the multivariate logistic regression. The analysis was carried out on the basis of the selection of the most significant variables and the imposition of the condition of non-correlation.

	Regression coefficients (B)	***Standard error***	***Wald test***	***P-value (<0.05)***	***Odds ratio (OR)***	***OR confidence interval 95%***	
						Lower	*Upper*
**Age**	0.023	0.051	0.204	0.652	1.023	0.926	1.130
$\frac{E_r}{BMI}$	0.269	0.311	0.748	0.387	1.309	0.711	2.410
**EBR**	-0.155	0.068	5.239	**0.022***	0.857	0.750	0.978
$\frac{E_l}{BMI}$	-0.100	0.251	0.158	0.691	0.905	0.553	1.480
$\frac{F_r}{BMI}$	-0.199	0.491	0.164	0.685	0.820	0.313	2.145
**FBR**	0.085	0.040	4.559	**0.033***	1.089	1.007	1.177
$\frac{F_l}{BMI}$	-0.011	0.321	0.001	0.972	0.989	0.527	1.855
**RUR**	0.032	0.122	0.068	0.794	1.032	0.813	1.312
**LUR**	-0.028	0.086	0.107	0.743	0.972	0.821	1.151

E_r: maximum force of right knee extensors; E_l: maximum force of left knee extensors; F_r: maximum force of right knee flexors; F_l: maximum force of left knee flexors; EBR: Extensor Bilateral Ratio; FBR: Flexor Bilateral Ratio; RUR: Right Unilateral Ratio; LUR: Left Unilateral Ratio. *: Statistically different values.

Table S2. Results of the Cox regression: multivariate survival analysis at 8 degrees of freedom.

	Regression coefficients (B)	*Standard error*	*Wald test*	*P-value (<0.05)*	*Hazard ratio (HR)*	*HR confidence interval 95%*	
						Lower	*Lower*
$\frac{E_r}{BMI}$	-0.17	0.96	0.03	0.86	0.84	0.13	-0.17
$\frac{E_l}{BMI}$	0.20	0.60	0.11	0.74	1.23	0.38	0.20
$\frac{F_r}{BMI}$	-0.22	0.94	0.06	0.81	0.80	0.13	-0.22
$\frac{F_l}{BMI}$	0.47	0.68	0.48	0.49	1.61	0.42	0.47
**FBR**	1.64	0.58	8.10	**0.01***	5.16	1.67	1.64
**EBR**	-12.41	506.96	0.00	0.98	0.00	0.00	-12.41
**RUR**	0.70	0.56	1.60	0.21	2.01	0.68	0.70
**LUR**	-0.01	0.67	0.01	0.99	0.99	0.27	-0.01

*: Statistically different values.

Table S3. Results of the ROC curves analysis. This method was implemented to maximize the sensitivity and specificity indices to derive optimized thresholds compared to those currently used by therapists.

Variables	*Current pathological cut-off*	*Sensitivity*	*Specificity*	*Optimized cut-off*	*Sensitivity*	*Specificity*	*Area under the curve (AUC)*	*P-value*
$\frac{E_r}{BMI}$	20	0.86	0.16	26.03	0.64	0.67	0.65	**0.02***
$\frac{E_l}{BMI}$	20	0.86	0.13	24.18	0.68	0.50	0.57	0.32
$\frac{F_r}{BMI}$	15	0.55	0.27	16.65	0.51	0.82	0.34	**0.02***
$\frac{F_l}{BMI}$	15	0.68	0.27	20.00	0.41	0.84	0.54	0.57
**EBR**	18	0.07	1.00	8.00	0.47	0.77	0.41	0.19
**FBR**	18	0.36	0.93	17.00	0.41	0.91	0.68	**0.01***
**RUR**	40	0.59	0.79	39.06	0.68	0.76	0.75	**0.01***
**LUR**	40	0.27	0.82	33.65	0.59	0.64	0.56	0.33

E_r: maximum force of right knee extensors; E_l: maximum force of left knee extensors; F_r: maximum force of right knee flexors; F_l: maximum force of left knee flexors; EBR: Extensor Bilateral Ratio; FBR: Flexor Bilateral Ratio; RUR: Right Unilateral Ratio; LUR: Left Unilateral Ratio. *: Statistically different values.
